# Changes in Serum Fatty Acid Composition and Metabolome-Microbiome Responses of Heigai Pigs Induced by Dietary N-6/n-3 Polyunsaturated Fatty Acid Ratio

**DOI:** 10.3389/fmicb.2022.917558

**Published:** 2022-06-22

**Authors:** Liyi Wang, Qiuyun Nong, Yanbing Zhou, Ye Sun, Wentao Chen, Jintang Xie, Xiaodong Zhu, Tizhong Shan

**Affiliations:** ^1^College of Animal Sciences, Zhejiang University, Hangzhou, China; ^2^Key Laboratory of Molecular Animal Nutrition, Ministry of Education, Zhejiang University, Hangzhou, China; ^3^Key Laboratory of Animal Feed and Nutrition of Zhejiang Province, Hangzhou, China; ^4^Shandong Chunteng Food Co. Ltd., Zaozhuang, China

**Keywords:** Heigai pig, polyunsaturated fatty acids, fatty acid composition, metabolome, microbiome

## Abstract

Changing fatty acid composition is a potential nutritional strategy to shape microbial communities in pigs. However, the effect of different n-6/n-3 polyunsaturated fatty acid (PUFA) ratios on serum fatty acid composition, microbiota, and their metabolites in the intestine of pigs remains unclear. Our study investigated the changes in serum fatty acid composition and metabolome–microbiome responses induced by dietary n-6/n-3 PUFA ratio based on a Heigai-pig model. A total of 54 Heigai finishing pigs (body weight: 71.59 ± 2.16 kg) fed with 3 types of diets (n-6/n-3 PUFA ratios are 8:1, 5:1, and 3:1) were randomly divided into 3 treatments with 6 replications (3 pigs per replication) for 75 days. Results showed that dietary n-6/n-3 PUFA ratio significantly affected biochemical immune indexes including glucose (Glu), triglycerides (TG), total cholesterol (TChol), non-esterified fatty acid (NEFA), high-density lipoprotein (HDL), low-density lipoprotein (LDL), and total thyroxine (TT4), and medium- and long-chain fatty acid composition, especially n-3 PUFA and n-6/n-3 PUFA ratio in the serum. However, no significant effects were found in the SCFAs composition and overall composition of the gut microbiota community. In the low dietary n-6/n-3 PUFA ratio group, the relative abundance of *Cellulosilyticum*, *Bacteroides*, and *Alloprevotella* decreased, *Slackia* and *Sporobacter* increased. Based on the metabolomic analysis, dietary n-6/n-3 PUFA ratio altered the metabolome profiles in the colon. Moreover, Pearson’s correlation analysis indicated that differential microbial genera and metabolites induced by different n-6/n-3 PUFA ratio had tight correlations and were correlated with the n-6 PUFA and n-3 PUFA content in longissimus dorsi muscle (LDM) and subcutaneous adipose tissue (SAT). Taken together, these results showed that lower dietary n-6/n-3 PUFA ratio improved serum fatty acid composition and metabolome–microbiome responses of Heigai pigs and may provide a new insight into regulating the metabolism of pigs and further better understanding the crosstalk with host and microbes in pigs.

## Introduction

Pork is one of the most produced and consumed meats in the world. It is an important source of protein, lipids, and other nutrients in the human diet, which is closely related to human health. However, pork has not reached the best fatty acid composition that is beneficial to human health due to the regular feeding practices (Dugan et al., 2015). Pig is monogastric, meaning that the fatty acid composition of pork closely reflects the fatty acid composition of the diet because dietary polyunsaturated fatty acids (PUFAs) do not undergo significant transformation in the gastrointestinal tract and fatty acids are also largely unmetabolized by microbes (Jaturasitha et al., 2016). In recent years, the type and composition of adding nutrients to diet have been reported to regulate host health and metabolism both in animals and humans (Petry et al., 2020; Wang et al., 2021). Many studies have demonstrated that microbiota is closely related to host metabolism and health (Tremaroli and Backhed, 2012; Diao et al., 2016). Especially, fatty acid composition in the diet was found to shape the microbial communities in pigs (Che et al., 2019; Lauridsen, 2020). PUFAs are straight-chain fatty acids with two or more double bonds and a carbon chain length of 18–22 carbon atoms that are closely related to human health and disease risk (Calder, 2015). Previous studies found PUFAs could regulate growth performance and pork quality both in lean breeds pigs and Chinese local fatty breeds pigs (De Tonnac and Mourot, 2018; Nong et al., 2020). However, there are few studies on the distribution of microorganisms and relevant metabolome in pigs fed with different n-6/n-3 PUFA ratios.

Fatty acids in the blood are often used as biomarkers to monitor dietary fat intake and disease risk (Bermudez-Cardona and Velasquez-Rodriguez, 2016). After feeding different fatty acid composition diets, measuring the biochemical immune indexes and fatty acid composition in the blood helps to monitor the effect of fatty acid absorption and inferring the effect on the health of the pig. However, there are few related reports on the changes in fatty acid composition in serum after feeding the diets with different n-6/n-3 PUFA ratios in pigs, especially in Chinese local pig breeds. Heigai pigs are one of the Chinese local fat-type pig breeds with the specific characteristics of farrowing rate, good meat quality, coarse feeding tolerance, strong disease resistance, and so on (Guo et al., 2016). Hence, it is worth studying the effects of dietary n-6/n-3 PUFA ratio on changes of serum fatty acid composition and metabolome–microbiome responses of Heigai pigs.

In this study, Heigai pigs were used to investigate the effects of feeding different n-6/n-3 PUFA ratios diets on fatty acid composition and biochemical immune indexes in serum and metabolome–microbiome responses in the intestine of Heigai pigs. Our findings will provide references for the nutritional regulation and metabolism of pigs and further understanding the crosstalk with host and microbes in pigs.

## Materials and Methods

### Experimental Design and Animal Sample Collection

The Zhejiang University Animal Care and Use Committee approved all procedures and housing (ZJU20170466). A total of 54 Heigai finishing pigs (body weight: 71.59 ± 2.16 kg) fed with 3 types of diets (n-6/n-3 PUFA ratios 8:1, 5:1, and 3:1) were randomly divided into 3 treatments with 6 replications (3 pigs per replication) with 12-h light/dark cycle, free access to water and food. The nutritional levels and PUFA composition of the diets are shown in Supplementary Tables 1, 2 as we previously reported (Nong et al., 2020). After 75 days of feeding (5 days preliminary feeding period and 70 days formal test period), 1 pig per replication was selected and fasted for 12 h, then humanely sacrificed. About 8 ml of serum were rapidly collected by standing, centrifuging, and absorbing supernatant from blood samples, and about 10 cm colonic samples were rapidly collected. All samples were frozen in liquid nitrogen immediately and subsequently stored at −80°C for further analysis.

### Medium- and Long-Chain Fatty Acid Composition Analysis

The fatty acid profile was identified and quantified in serum referring to previous studies (Kouba et al., 2003; Zhou et al., 2019). About 200 μL samples were taken and mixed with 5 mL methylene dichloride–methanol (2:1 v/v), mixed for 2 min followed by shaking for 20 min. Free fatty acids were extracted twice with hexane and collected for derivatization to methyl esters (FAME). The supernatant was taken for detection using gas chromatograph to quantify fatty acid methyl esters (GC-MS, infusion volume 1 μL, split ratio 10:1, split injection) and compared with standards to identify and quantify medium and long-chain fatty acid. The chromatograms were processed and calculated by Varian-Star software. Fatty acid content was shown as a percentage of total fatty acids.

### Serum Short-Chain Fatty Acids Composition Analysis

About 200 μL samples were taken and mixed with 50 μL 50% phosphoric acid in a 2-mL centrifuge tube for 2 min. A total of 100 μL isopropyl ether solution (contains 5 ug/mL 4-methylvaleric acid) was added and homogenized for 1 min, centrifuged for 20 min (4°C, 14,000 rpm/min), and allowed to stand for 30 min. Then SCFAs were identified and quantified using GC-MS as the same method described above (identify and quantify medium and long-chain fatty acid). SCFAs contents were shown as a percentage of total fatty acids.

### Determination of Biochemical Immune Indexes

The contents of glucose (Glu), triglycerides (TG), total cholesterol (TChol), non-esterified fatty acid (NEFA), high-density lipoprotein (HDL), low-density lipoprotein (LDL), cortisol, immunoglobulin G (IgG), and total thyroxine (TT4) in serum were measured by the Second People’s Hospital of Hangzhou province.

### DNA Extraction, 16S rRNA Gene Amplification, and Sequencing

DNA from different samples in the colon was extracted using the E.Z.N.A. ^®^Stool DNA Kit (D4015, Omega, Inc., United States) according to the manufacturer’s instructions. The total DNA was stored at −80°C until measurement in the PCR by LC-Bio Technology Co., Ltd, Hang Zhou, Zhejiang Province, China. 16S rRNA genes of the V3–V4 region of the bacteria were amplified by PCR by using primers 341F (5′-CCTACGGGNGGCWGCAG-3′) and 805R (5′-GACTACHVGGGTATCTAATCC-3′) (Logue et al., 2016). PCR amplification was performed in a total volume of 25 μL reaction mixture and the PCR products were confirmed with 2% agarose gel electrophoresis and purified by AMPure XT beads (Beckman Coulter Genomics, Danvers, MA, United States) and quantified by Qubit (Invitrogen, United States).

Samples were sequenced on an Illumina NovaSeq platform and paired-end reads were merged using FLASH. Quality filtering on the raw reads was performed under specific filtering conditions to obtain the high-quality clean tags according to the fqtrim (v0.94). Chimeric sequences were filtered using V search software (v2.3.4). After dereplication using DADA2, we obtained the feature table and feature sequence. Alpha diversity and beta diversity were calculated by normalizing to the same sequences randomly. Then according to SILVA (release 132) classifier, feature abundance was normalized using the relative abundance of each sample. Alpha diversity is applied for analyzing the complexity of species diversity for a sample through 5 indices, including Chao1, Observed species, Goods coverage, Shannon, Simpson, and all this indices in our samples were calculated with QIIME2. Beta diversity was calculated by QIIME2, the graphs were drawn by R package. Blast was used for sequence alignment, and the feature sequences was annotated with SILVA database for each representative sequence. Other diagrams were implemented using the R package (version 4.0.5).

### Untargeted Metabolomics Relative-Quantitative Analysis

Colonic samples were stored overnight at −20°C for metabolite detection according to the manufacture’s standard protocol (Yin et al., 2020). Briefly, samples were acquired by using the LC-MS system and were performed by a Thermo Scientific UltiMate 3000 HPLC followed by machine orders. Chromatographic separation conditions were as fillowa: column temperature: 35°C; flow rate: 0.4 mL/min. Using TripleTOF6600plus (SCIEX, Framingham, MA, United States), metabolites eluted from the column were detected. Raw data files were converted into mzXML format and then processed by the XCMS, CAMERA, and metaX, and the intensity of peak data was further preprocessed by metaX (Li et al., 2018). Principle component analysis (PCA) was performed for outlier detection and batch effects evaluation by using the pre-processed dataset. Supervised PLS-DA was conducted through metaX to discriminate the different variables between different n-6/n-3 PUFA ratio groups. The differential metabolites were defined by variable importance projection (VIP) values above 1.0 and *P* < 0.05. In addition, commercial databases including Kyoto Encyclopedia of Genes and Genomes (KEGG) were used to determine which differentially expressed genes (DEGs) were significantly enriched in metabolic pathways. Enriched pathways and terms were visualized by centupled and metaplot function.

### Statistical Analysis

One-way ANOVA in SPSS 20.0 (IBM-SPSS Inc., Chicago, IL, United States) software was used to analyze the data. Multiple comparisons between groups were applied using Duncan *post-hoc* test and accepted as significant if *P* < 0.05. Pearson correlation was analyzed to evaluate the correlation between bacterial genera and meat quality, fatty acid profiles, biochemical indexes in serum, and metabolites. Data visualization and statistical analyses were performed using the GraphPad Prism 6.0.2 software package (Monrovia, CA, United States) and R software (version 4.0.5).

## Results

### Dietary N-6/n-3 Polyunsaturated Fatty Acid Ratio Affected Biochemical Immune Indexes in Serum of Heigai Pigs

**FIGURE 1:**
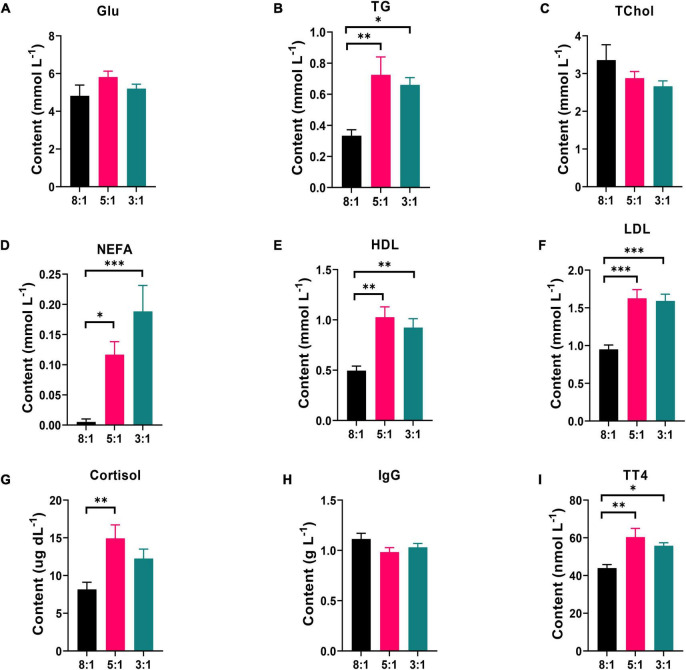
Dietary n-6/n-3 PUFA ratio affects biochemical indexes in serum of Heigai Pigs. The contents of glucose [Glu, **(A)**], triglyceride [TG, **(B)**], total cholesterol [TChol, **(C)**], non-esterified fatty acid [NEFA, **(D)**], high-density lipoprotein [HDL, **(E)**], low-density lipoprotein [LDL, **(F)**], cortisol **(G)**, immunoglobulin G [IgG, **(H)**], and total thyroxine [TT4, **(I)**] in serum from Heigai pigs fed with different diets. 8:1, 5:1, 3:1: different n-6/n-3 PUFA ratio diets. Data are presented as mean ± SEM (*n* = 6). *P < 0.05, **P < 0.01, ***P < 0.001.

The changes in biochemical immune indexes in serum (including Glu, TG, TChol, NEFA, HDL, LDL, cortisol, IgG, and TT4) are shown in Figure 1. The pigs fed the dietary n-6/n-3 PUFA ratio of 8:1 contained the lower level of HDL (Figure 1E) and LDL (Figure 1F) than 5:1 (*P* < 0.01) and 3:1 (*P* < 0.01) in serum. Compared with the pigs fed the dietary n-6/n-3 PUFA ratio of 5:1, lower TG (Figure 1B, *P* < 0.01), NEFA (Figure 1D, *P* < 0.05), cortisol (Figure 1G, *P* < 0.01), and TT4 (Figure 1I, *P* < 0.01) were found in ratio of 8:1 group. Similarly, 3:1 group had higher contents of (Figure 1B, *P* < 0.05), NEFA (Figure 1D, *P* < 0.01) and TT4 (Figure 1I, *P* < 0.05) than 8:1 group. However, the content of Glu, TChol, and IgG had no significant differences in the serum of Heigai pigs (Figures 1A,C, and 1 H, *P* > 0.05).

### Dietary N-6/n-3 Polyunsaturated Fatty Acid Ratio Induced Alterations in Fatty Acid Profiles of Serum in Heigai Pigs

To analyze the effects of dietary n-6/n-3 PUFA ratio on fatty acid profiles of serum, we measured the changes in the compositions of fatty acids, including medium-chain and long-chain fatty acid and SCFAs (Tables 1, 2). As shown in Table 1, the decreased dietary n-6/n-3 PUFA ratio led to a significant decrease in arachidonic acid [C20:4 (n-6)] and docosahexaenoic acid [C22:4 (n-6)]. Pigs fed with the dietary n-6/n-3 PUFA ratio of 5:1 had higher levels of palmitic acid (C16:0, *P* < 0.05) and heptadecenoic acid [C17:1 (n-7), *P* < 0.01] than the 3:1 group. Dietary n-6/n-3 PUFA ratio of 8:1 had the lower percentages of α-linolenic acid [C18:3 (n-3), *P* < 0.01] and eicosapentaenoic acid [C20:5 (n-3), *P* < 0.05]. Compared with the dietary n-6/n-3 PUFA ratio of 5:1 and 3:1 group, pigs of the 8:1 group had ta lower n-3 PUFA (*P* < 0.01) and a higher n-6/n-3 PUFA ratio (*P* < 0.01). There was no significant difference in the remainder of the fatty acid (*P* > 0.05) after feeding different dietary n-6/n-3 PUFA ratios. Besides, the changes in SCFAs composition in serum are shown in Table 2. The content of acetic acid in the serum of the three groups was the highest and isobutyric acid ranked second. The sum of acetic acid and isobutyric acid exceeds 90% of the total SCFAs. However, all of the SCFAs contents were not significantly affected by different n-6/n-3 PUFA ratio diets (*P* > 0.05).

**TABLE 1:** Medium- and long-chain fatty acid profiles in serum of Heigai pigs fed with different diets.

Item	Values of each group (%)^1^			SEM	*p*-Value
	8:1	5:1	3:1		
C8:0	0.77	0.48	0.79	0.09	0.29
C10:0	0.28	0.23	0.15	0.03	0.15
C14:0	0.27	0.41	0.21	0.04	0.12
C14:1 (n-5)	0.25	0.15	0.11	0.03	0.15
C16:0	11.10^ab^	13.65^a^	7.69^b^	1.13	0.09
C16:1	0.48	0.51	0.24	0.06	0.15
C17:0	0.35	0.47	0.29	0.04	0.15
C17:1 (n-7)	0.13^AB^	0.18^A^	0.09^B^	0.01	0.02
C18:0	13.72	14.28	11.05	1.03	0.42
C18:1 (n-9)	9.72	11.05	7.48	0.80	0.19
C18:2 (n-6)	8.71	11.75	8.85	0.79	0.21
C18:3 (n-6)	0.20	0.19	0.09	0.02	0.12
C18:3 (n-3)	0.60^B^	3.01^A^	2.80^A^	0.35	<0.01
C20:2 (n-6)	0.37	0.40	0.33	0.03	0.62
C20:3 (n-6)	0.49	0.43	0.35	0.04	0.35
C20:4 (n-6)	8.47^a^	6.65^ab^	5.04^b^	0.66	0.10
C20:5 (n-3)	0.38^b^	1.24^a^	1.12^a^	0.14	0.02
C22:1 (n-9)	0.19	0.21	0.11	0.03	0.35
C22:4 (n-6)	0.90^Ac^	0.43^ABd^	0.27^B^	0.10	0.01
C22:5 (n-6)	2.22	3.31	3.70	0.37	0.24
C24:0	39.02	29.74	47.20	4.39	0.28
C22:6 (n-3)	0.84	0.77	1.23	0.12	0.25
C24:1 (n-9)	0.56	0.47	0.82	0.10	0.34
SFA	65.98	59.76	67.62	2.38	0.39
UFA	34.02	40.24	32.38	2.38	0.39
PUFA	23.17	28.17	23.78	1.73	0.46
MUFA	10.85	12.06	8.60	0.76	0.17
n-6 PUFA	21.35	23.15	18.63	1.56	0.52
n-3 PUFA	1.81^B^	5.02^A^	5.15^A^	0.45	<0.01
n-6: n-3 PUFA	11.81^A^	4.73^B^	3.62^B^	1.06	<0.01

*^1^SEM, standard error of the mean. (n = 6, number of replicates).*

*^a, b, c, d^Different superscripts within a row indicate significant differences (p < 0.05).*

*^A, B^Different superscripts within a row indicate significant differences (p < 0.01).*

*SFA: saturated fatty acid, SFA = Σ (C8:0, C10:0, C14:0, C16:0, C17:0, C18:0, C24:0); UFA: unsaturated fatty acid, UFA = Σ (C14:1 (n-5), C16:1, C17:1 (n-7), C18:1 (n-9), C18:2 (n-6), C18:3 (n-6), C18:3 (n-3), C20:2 (n-6), C20:3 (n-6), C20:4 (n-6), C20:5 (n-3), C22:1 (n-9), C22:4 (n-6), C22:5 (n-6), C22:6 (n-3), C24:1 (n-9)); MUFA: monounsaturated fatty acid, MUFA = Σ (C14:1 (n-5), C16:1, C17:1 (n-7), C18:1 (n-9), C22:1 (n-9), C24:1 (n-9)); PUFA: polyunsaturated fatty acid, PUFA = Σ (C18:2 (n-6), C18:3 (n-6), C18:3 (n-3), C20:2 (n-6), C20:3 (n-6), C20:4 (n-6), C20:5 (n-3), C22:4 (n-6), C22:5 (n-6), C22:6 (n-3)). n-6 PUFA = Σ (C18:2 (n-6), C18:3 (n-6), C20:2 (n-6), C20:3 (n-6), C20:4 (n-6), C22:4 (n-6), C22:5 (n-6)). n-3 PUFA = Σ (C18:3 (n-3), C20:5 (n-3), C22:6 (n-3)). C16:1 = Σ (C16:1(n-7), C16:1(n-9)). 8:1, 5:1, 3:1: different n-6/n-3 PUFA ratio diets.*

**TABLE 2:** Short chain fatty acid composition in serum of Heigai pigs fed with different diets.

Item	Values of each group (%)^1^			SEM^2^	*p*-Value
	8:1	5:1	3:1		
Acetic acid	74.81	76.85	76.15	0.98	0.72
Propionic acid	0.99	0.90	1.22	0.10	0.45
Butyric acid	1.65	1.48	1.60	0.05	0.40
Isobutyric acid	15.92	14.61	14.37	0.90	0.77
Valeric acid	1.06	0.79	1.01	0.07	0.25
Isovaleric acid	0.72	0.66	0.77	0.02	0.19
Hexanoic acid	4.86	4.71	4.90	0.06	0.39

*^1^8:1, 5:1, 3:1: different n-6/n-3 PUFA ratio diets.*

*^2^SEM, standard error of the mean.*

*(n = 6, number of replicates).*

### Dietary N-6/n-3 Polyunsaturated Fatty Acid Ratio Changed Bacterial Community Composition in Colonic Digesta of Heigai Pigs

**FIGURE 2:**
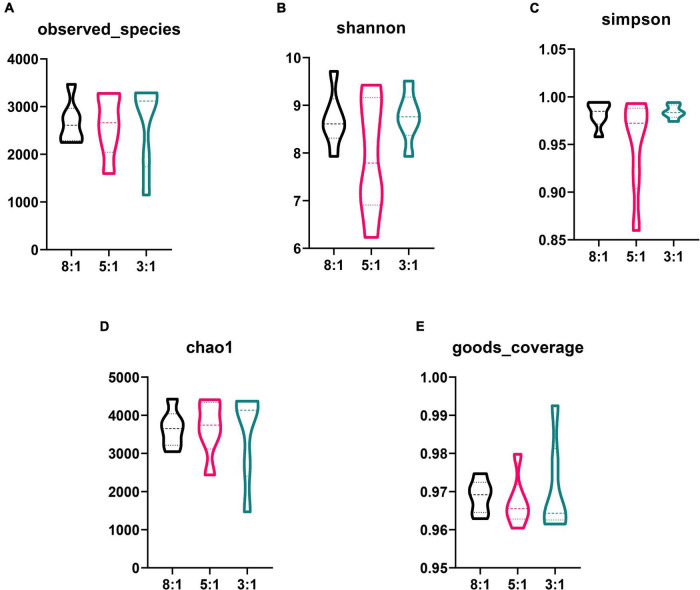
Differences in the colonic bacterial α-diversity index after different n-6/n-3 PUFA ratio treatment. 8:1, 5:1, 3:1: different n-6/n-3 PUFA ratio diets. Data are presented as mean ± SEM (*n* = 5).

**FIGURE 3:**
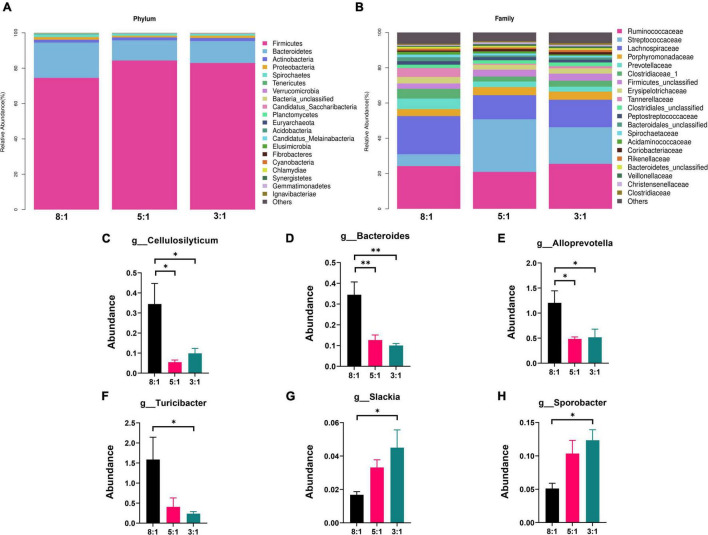
The relative abundance of bacteria in colonic digesta of Heigai pigs at the phylum, family and genus level. **(A)** Differential bacteria in colonic digesta at the phylum level. **(B)** Differential bacteria in colonic digesta at the family level. **(C–H)** The relative abundances of bacteria in colonic digesta at the genus level. 8:1, 5:1, 3:1: different n-6/n-3 PUFA ratio diets. Data are presented as mean ± SEM (*n* = 5). *P < 0.05, ^**^P < 0.01.

In total, 855,978 clean sequences were acquired after data filtering by using a normal quality control system. These clean sequences were clustered into 5,874 operational taxonomic units (OTUs) by using the standard of 97% similarity level. With the increase in sampling quantity, the rarefaction curve gradually flattened out (Supplementary Figure 1). The alpha-diversity indexes of the colonic bacterial community are presented in Figure 2. There is no significant difference in the observed species index, Shannon index, Simpson index, chao1 index, and goods coverage index among the three groups (*P* > 0.05). Also, the PCA plot indicated that different n-6/n-3 PUFA ratios could not distinguish the panorama of the colonic bacterial community (Supplementary Figure 2). The stacked bar chart represented the top 20 phyla in the three groups, and the top 2 phyla were *Firmicutes* and *Bacteroidetes* (Figure 3A). Compared with the 8:1 group, the 5:1 and 3:1 groups had higher contents of *Firmicutes* and lower *Bacteroidetes* (Supplementary Figure 3A). At the family level, the stacked bar chart represented the top 20 differential microorganisms in the three groups and the top 3 were *Ruminococcaceae*, *Streptococcaceae*, and *Lachnospiraceae* (Figure 3B and Supplementary Figure 3B). At the genus level, the 8:1 group had the higher relative abundances of *Cellulosilyticum* (*P* < 0.05), *Bacteroides* (*P* < 0.01), and *Alloprevotella* (*P* < 0.05) compared with the 5:1 and 3:1 group (Figures 3C–E). Pigs fed with n-6/n-3 PUFA ratio of 3:1 had less *Turicibacter* abundances but more *Slackia* and *Sporobacter* abundances than n-6/n-3 PUFA ratio of the 8:1 group in colonic digesta (Figures 3F–H, *P* < 0.05).

### Dietary N-6/n-3 Polyunsaturated Fatty Acid Ratio Altered Metabolites in Colonic Digesta of Heigai Pigs

**FIGURE 4:**
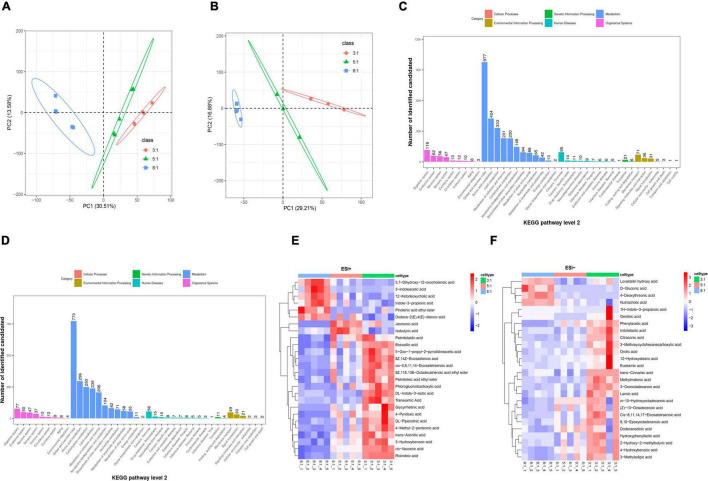
The effects of dietary n-6/n-3 PUFA ratio on colonic metabolites. **(A)** Principal coordinates analysis (PCA) plot of colonic metabolites (ESI +) of Heigai pigs in different groups. **(B)** PCA plot of colonic metabolites (ESI-) of Heigai pigs in different group. The ellipse represents the 95% confidence interval of each group. **(C,D)** Kyoto Encyclopedia of Genes and Genomes (KEGG) pathway enrichment of these significant differential metabolites identified among three different groups under ESI + and ESI- modes. **(E,F)** Heatmap of colonic metabolites under ESI + and ESI- modes in each group. Only metabolites with *P* < 0.05 are displayed.

As shown in Figures 4A,B the PCA plot revealed an obvious and regular variation between the three n-6/n-3 PUFA ratio groups in both ESI- and ESI + modes. KEGG pathway enrichment analysis demonstrated that these differential features were mostly enriched in metabolism, including global and overview maps, amino acid metabolism, and lipid metabolism in ESI + mode (Figure 4C). Metabolism, such as global and overview maps, amino acid metabolism, and carbohydrate metabolism, pathways were enriched in ESI + mode (Figure 4D). A total of 803 metabolites in the ESI + mode and 542 in the ESI-mode were identified using the LC-MS metabolomic technique. Heatmap showed the differential features with *P* < 0.05 (Figures 4E,F). With increasing n-6/n-3 PUFA ratio in the diet, the concentrations of 12-ketodeoxycholic acid and indole-3-propionic acid were decreased, but the concentrations of palmitelaidic acid, eicosatetraenoic acid, and *cis-*vaccenic acid were increased under ESI + mode (Figure 4E and Supplementary Figure 4A). The contents of D-gluconic acid and 4-deoxythreonic acid were decreased but the contents of 9,10-epoxy octadecanoic acid, dodecanedioic acid, and 4-hydroxybenzoic acid were increased in colonic digesta after feeding low n-6/n-3 PUFA ratio diet under ESI- mode (Figure 4F and Supplementary Figure 4B).

### Correlations Between Bacterial Genera and Meat Quality, Fatty Acid Profiles, Biochemical Indexes in Serum, and Metabolites in Colonic Digesta of Heigai Pigs

**FIGURE 5:**
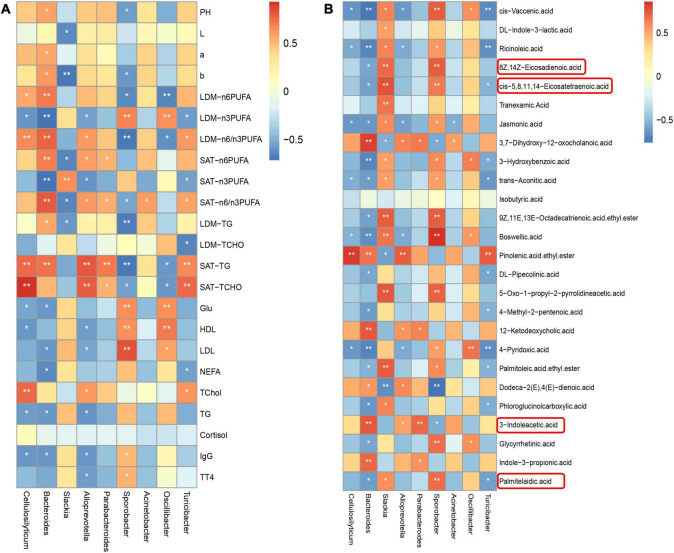
Correlations among meat quality, fatty acid profiles, the biochemical indexes in serum, differential microbiota at the genus level and bacterial metabolites in colonic digesta of Heigai pigs. **(A)** Correlations between meat quality, fatty acid profiles, the biochemical indexes in serum with differential microbiota at the genus level of Heigai pigs. **(B)** Correlations between meat quality, fatty acid profiles, the biochemical indexes in serum with bacterial metabolites in colonic digesta of Heigai pigs. Each square represents a Pearson correlation coefficient between a genus and an index, while the gradation of color represents the size of each correlation coefficient. The red color represents a positive correlation, while the blue color represents a negative correlation. pH, pH 45 min–pH 24 h; L, L 24 h–L 45 min; a, a 24 h–a 45 min; b, b 24 h–b 45 min. *P < 0.05, **P < 0.01.

Based on our previous study on meat quality and fatty acid profile in longissimus dorsi muscle (LDM) and subcutaneous adipose tissue (SAT) (Nong et al., 2020), we found that at the genus level, the differential microbiota showed a significant correlation with meat quality, fatty acid profile, and the biochemical index in serum based on the Pearson correlation analysis (Figure 5A). *Cellulosilyticum* was positively correlated with n-6 PUFA content (*P* < 0.05) and n-6/n-3 PUFA ratio (*P* < 0.01) in LDM, TG, and TChol content in SAT (*P* < 0.01) and TChol content in serum (*P* < 0.01), while it was negatively correlated with n-3 PUFA content in LDM (*P* < 0.05), Glu, HDL, TG, and IgG content in serum (*P* < 0.05). *Bacteroides* was positively correlated with ΔpH (*P* < 0.05), Δa (*P* < 0.05), Δb (*P* < 0.05), n-6 PUFA content, and n-6/n-3 PUFA ratio in LDM and SAT (*P* < 0.01), and TG content in LDM (*P* < 0.05) and SAT (*P* < 0.01), while it was negatively correlated with n-3 PUFA content in LDM and SAT (*P* < 0.01), Glu, LDL, NEFA, TG and IgG content in serum (*P* < 0.05). *Alloprevotella* was positively correlated with n-6/n-3 PUFA ratio in LDM (*P* < 0.05), n-6 PUFA content (*P* < 0.05), n-6/n-3 PUFA ratio (*P* < 0.05), TG (*P* < 0.01) and TChol (*P* < 0.01) in SAT, and TChol (*P* < 0.05) in serum, whereas it was negatively correlated with n-3 PUFA in LDM and SAT (*P* < 0.05) and HDL, LPL, TG, IgG, and TT4 (*P* < 0.05) in serum. *Turicibacter* was positively correlated with n-6/n-3 PUFA ratio in LDM and SAT (*P* < 0.05), TG and TChol in SAT (*P* < 0.01), TChol in serum (*P* < 0.05), whereas it was negatively correlated with n-3 PUFA in LDM and SAT (*P* < 0.05), TChol in LDM (*P* < 0.05), and NEFA in serum (*P* < 0.05). *Slackia* was positively correlated with n-3 PUFA in SAT (*P* < 0.01), while it was negatively correlated with ΔL (*P* < 0.05), Δb (*P* < 0.01), n-6 PUFA content, n-6/n-3 PUFA ratio in SAT (*P* < 0.05) and TG content in LDM (*P* < 0.05). *Sporobacter* was positively correlated with n-3 PUFA in LDM (*P* < 0.01) and Glu (*P* < 0.01), HDL (*P* < 0.01), LPL (*P* < 0.01), IgG, and TT4 in serum (*P* < 0.05), whereas it was negatively correlated with ΔpH (*P* < 0.05), Δb (*P* < 0.05), n-6 PUFA content (*P* < 0.05), n-6/n-3 PUFA ratio (*P* < 0.01), and TG (*P* < 0.01) in LDM, n-6/n-3 PUFA ratio (*P* < 0.01), TG (*P* < 0.01), and TChol (*P* < 0.05) in SAT.

As shown in Figure 5B, at the genus level, the differential microbiota presented a significant correlation with metabolites in the colonic digesta based on the Pearson correlation analysis. 12-Ketodeoxycholic acid was positively correlated with *Bacteroides* (*P* < 0.01) and *Alloprevotella* (*P* < 0.05). *cis-*Vaccenic acid was positively correlated with *Slackia* (*P* < 0.05) and *Sporobacter* (*P* < 0.01), whereas it was negatively correlated with *Cellulosilyticum* (*P* < 0.05), *Bacteroides* (*P* < 0.01), *Alloprevotella* (*P* < 0.05), and *Turicibacter* (*P* < 0.01). Eicosatetraenoic acid was positively correlated with *Slackia* and *Sporobacter* (*P* < 0.01), while it was negatively correlated with *Bacteroides* (*P* < 0.05). Indole-3-propionic acid was positively correlated with *Slackia* (*P* < 0.05). Palmitelaidic acid was positively correlated with *Slackia* (*P* < 0.05) and *Sporobacter* (*P* < 0.01), whereas it was negatively correlated with *Bacteroides* and *Turicibacter* (*P* < 0.05).

## Discussion

Plasma and serum are closely associated with dietary fat intake and are rich sources of information regarding health state (Geyer et al., 2019). In this study, we found that as the ratio of n-6/n-3 PUFA decreased, the contents of TG, NEFA, HDL, LDL, and TT4 significantly increased, while TChol had a decreased tendency. Our results are inconsistent with a previous study in a mouse model, which found that a n-3 PUFA-rich diet decreased HDL and had no effect on TG, TChol, and LDL contents in serum (Tian et al., 2018). It may be because the PUFA diet has different effects on different species. Besides, the TG contents in serum are inconsistent with our previous findings in LDM and SAT (Nong et al., 2020). This might be because a low n-6/n-3 PUFA diet may reduce TG deposition in muscle and adipose tissue and enhance TG release, resulting in increase in serum TG content. Although the low n-6/n-3 PUFA ratio diet increased NEFA contents in serum, they are still within the normal range. The thyroid hormone regulates crucial biological functions involving energy, thermogenesis, development, and growth, and T4 is one of the important thyroid hormones (Salvatore et al., 2014). Our results showed that a low n-6/n-3 PUFA ratio diet could promote the production of TT4 and may regulate the metabolism of various body systems. However, the specific mechanism needs to be further explored. In addition, we found that different n-6/n-3 PUFA ratios in the diet affected medium- and long-chain fatty acid composition in serum. As dietary n-6/n-3 PUFA ratio decreased, the n-6 PUFA including arachidonic acid and docosahexaenoic acid decreased, the total n-3 PUFA increased and n-6/n-3 PUFA decreased in serum. These results are consistent with a previous study on lean-type pigs, which investigated that the n-6/n-3 PUFA ratio of serum decreases with a decreasein dietary n-6/n-3 PUFA ratio, while the content of n-3 PUFA is the opposite (De Tonnac and Mourot, 2018). It is widely recognized that n-3 and n-6 PUFAs are closely related to human health, and people expect for high n-3 PUFA content and low n-6/n-3 PUFA ratio diet (O’connell et al., 2017; Lee et al., 2018). Hence, dietary fatty acid changes the fatty acid composition in serum and other tissues, making pork a good candidate for better fatty acid composition through dietary regulation (Li et al., 2015). Improving the fatty acid composition in the blood and tissues of pigs is help for health of pigs and producing n-3 PUFA-rich pork. Moreover, we can predict whether the ratio of fatty acids in the diet has reached the level of changing the fatty acid composition of meat through blood testing.

However, SCFAs composition in serum had no difference. SCFAs are produced by the intestinal microbial ferment dietary fibers and resistant starch. Previous research demonstrated that plasma and colonic SCFAs are related to metabolic activity and are associated with diseases (Hu et al., 2018). Besides, SCFAs could influence the health of the intestinal microbial community (Den Besten et al., 2013). In this study, we found the overall composition of the colonic microbiota communities was not altered among different groups. This result indicated that dietary n-6/n-3 PUFA ratio could not change the richness and evenness of the microbial community. Studies showed that dietary fat and fiber regulated the gut microbiota and its metabolites directly or indirectly (Hu et al., 2018; Zmora et al., 2019). Dietary fiber and high-fat diets have the greatest impact on SCFAs; however, n-3 and n-6 PUFA are more closely related to inflammation (Shen et al., 2014; Candido et al., 2018; De Caro et al., 2019). The dietary fatty acid used in the current experiment might not reach the level of changing the metabolism of SCFAs.

Recent studies have reported that the host and the intestinal microbiota have tight interactions to regulate health and homeostasis, which mainly expressed that microbiota plays a crucial role in host metabolism, such as the metabolism of amino acid, carbohydrate, lipid, nucleotide, and vitamin and host take nutrients in the intestine could affect the gut microbiota meanwhile (Shin et al., 2019; Meng et al., 2020). Although there is no change in microbial diversity in our study, the dietary n-6/n-3 PUFA ratio significantly changed the relative abundance of some taxa at the phylum, family, and genus levels. Lower n-6/n-3 PUFA ratio increased the abundance of *Firmicutes* and decreased the abundance of *Bacteroidetes*. *Firmicutes* and *Bacteroidetes* are the predominant phyla in the gut microbiome of most mammals and are related to metabolic syndrome like obesity and diabetes (Magne et al., 2020; Grigor’eva, 2021). At the family level, low n-6/n-3 PUFA ratio decreased the abundance of *Lachnospiraceae*. *Lachnospiraceae* is the main producer of SCFAs, is associated with intestinal diseases, and has been reported that increase in obese subjects (Zhao et al., 2019; Vacca et al., 2020). Hence, we thought that a lower n-6/n-3 PUFA ratio in diet may affect gut metabolism and lipid metabolism. Additionally, at the genus level, as n-6/n-3 PUFA ratio in diet decreases, the abundance of *Cellulosilyticum*, *Bacteroides*, and *Alloprevotella* significantly decreases. *Cellulosilyticum* has been reported to be associated with breaking down both fiber and protein (Jo et al., 2021). *Bacteroides*, which is reported to metabolize polysaccharides and oligosaccharides and provide nutrition and vitamins to the host and other intestinal microbial residents (Zafar and Saier, 2021). *Alloprevotella* has been reported to produce succinate and acetate promoting the gut barrier and exhibiting anti-inflammatory function (Downes et al., 2013). We found TG content in serum was negatively correlated with the abundance of *Cellulosilyticum*, *Bacteroides*, and *Alloprevotella*. It is inconsistent with a previous study in Chinese Erhualian pigs, which concluded the bacteria serum lipids had negative correlations with *Lactobacillus* and *Bacilli* (Huang et al., 2017). Besides, we discovered the *Cellulosilyticum*, *Bacteroides*, and *Alloprevotella* had significantly correlation with the contents of n-6 PUFA and n-3 PUFA in LDM and SAT which mainly presented that these taxa were positively correlated with n-6 PUFA and n-6/n-3 PUFA ratio but were negatively correlated with n-3 PUFA (Nong et al., 2020). Additionally, the n-6/n-3 PUFA ratio of 3:1 group had a higher abundance of *Slackia* and *Sporobacter*. *Slackia* has been suggested to play an important role in host lipid and xenobiotic metabolism (Cho et al., 2016) and the lower abundance of *Sporobacter* has been reported in patients with immune-mediated inflammatory diseases (Forbes et al., 2018). These results indicated that dietary n-6/n-3 PUFA ratio might mediate these genera, following affect the metabolism of fatty acid, which lead to the changes of fatty acid composition in serum and protect gut metabolism and health.

The results obtained in this study indicated that dietary n-6/n-3 PUFA ratio significantly altered metabolites in colonic digesta. We found indole-3-propionic acid decreased after feeding a lower n-6/n-3 PUFA ratio diet and it was positively correlated with *Bacteroides*. Indole-3-propionic acid is a gut microbiota-produced metabolite, which is a potential biomarker for the development of type 2 diabetes (T2D) (De Mello et al., 2017). Hence, the n-6/n-3 PUFA ratio diet may regulate glucolipid metabolism in Heigai pigs. As n-6/n-3 PUFA ratio in diet increases, some n-6 PUFA such as eicosatetraenoic acid increases and it was positively correlated with *Slackia* and *Sporobacter*. Eicosatetraenoic acid is a kind of biological active substance, which plays an important role in regulating the metabolism of lipid protein (Guan et al., 2021). These results indicated that the lower n-6/n-3 PUFA ratio could regulate the absorption and release of PUFA and protect gut health in pigs. Besides, palmitoleic acid and 4-hydroxybenzoic acid were increased in colonic digesta after feeding a lower n-6/n-3 PUFA ratio diet. A previous study showed that 4-hydroxybenzoic acid could inhibit the growth of most of the bacteria and yeasts at concentrations of 200–400 μg (Cho et al., 1998). Palmitoleic acid promotes the uptake of glucose in the body and is related to higher insulin concentration (Frigolet and Gutierrez-Aguilar, 2017). These results suggest that the lower n-6/n-3 PUFA ratio diet may affect glucose absorption, regulate glucolipid metabolism, inhibit bacteria growth and protect gut health in pigs.

## Conclusion

In conclusion, our current study demonstrated that dietary n-6/n-3 PUFA ratio regulated biochemical immune indexes and improved fatty acid composition, which mainly presented that dietary n-6/n-3 PUFA ratio significantly changed the relative abundance of genera including *Cellulosilyticum*, *Bacteroides*, *Alloprevotella*, *Slackia*, and *Sporobacter*. Moreover, the correlation results suggested that differential microbial genera and metabolites induced by different n-6/n-3 PUFA ratios were correlated with the n-6 and n-3 PUFA in LDM and SAT which indicated that the gut microbiota and metabolites might mediate the positive effect of lower n-6/n-3 PUFA ratio on the fatty acid composition of pork. These results may provide a new insight into regulating the metabolism of pig and further understanding the crosstalk with host and microbes in pigs. However, the potential molecular mechanism and risks of n-6/n-3 PUFA ratio on the health and metabolism of pigs are worth further study.

## Data Availability Statement

The data presented in this study are openly available in NCBI at SRA data and Metabolight under reference numbers PRJNA786967 and MTBLS4857.

## Ethics Statement

The animal study was reviewed and approved by the Zhejiang University Animal Care and Use Committee.

## Author Contributions

TS, LW, and QN designed the experiments, and wrote the manuscript, and analyzed the data. LW, QN, YZ, YS, and WC conducted the experiments. JX and XZ provide materials and sources. All authors have read and approved the final manuscript.

## Conflict of Interest

JX and XZ were employees of Shandong Chunteng Food Co. Ltd. (Zaozhuang, Shandong, China). The remaining authors declare that the research was conducted in the absence of any commercial or financial relationships that could be construed as a potential conflict of interest.

## Publisher’s Note

All claims expressed in this article are solely those of the authors and do not necessarily represent those of their affiliated organizations, or those of the publisher, the editors and the reviewers. Any product that may be evaluated in this article, or claim that may be made by its manufacturer, is not guaranteed or endorsed by the publisher.
