# Supplementary material for: Changes in Serum Fatty Acid Composition and Metabolome-Microbiome Responses of Heigai Pigs Induced by Dietary N-6/n-3 Polyunsaturated Fatty Acid Ratio
Source: Front Microbiol. 2022 Jun 22;13:917558. doi: 10.3389/fmicb.2022.917558 (PMC9257074; doi:10.3389/fmicb.2022.917558)
Supplement: Supplementary file 1 [file data_Sheet_1.docx]

***Supplementary Materials***

- 1. **Supplementary Tables**

**Table S1.** Ingredient composition and nutritional levels of the diet (air-dry basis)^1^.

| **Ingredient** | **Content (%)** | | | **Nutritional Levels**^2^ | **Content (%)** |
| --- | --- | --- | --- | --- | --- |
|  | **8:1** | **5:1** | **3:1** ^1^ |  |  |
| Corn | 50.00 | 50.00 | 50.00 | Crude protein | 11.85 |
| Soybean meal | 4.00 | 4.00 | 4.00 | Crude fiber | 12.20 |
| Wheat bran | 17.00 | 17.00 | 17.00 | Crude fat | 5.63 |
| Calcium hydrogen phosphate | 0.50 | 0.50 | 0.50 |  |  |
| Limestone powder | 1.00 | 1.00 | 1.00 |  |  |
| Dried ground hay | 23.70 | 23.70 | 23.70 |  |  |
| Soybean oil | 2.30 | 1.50 | 0.15 |  |  |
| Linseed oil | 0.70 | 1.50 | 2.85 |  |  |
| Salt | 0.30 | 0.30 | 0.30 |  |  |
| Premix | 0.50 | 0.50 | 0.50 |  |  |
| Digestive energy, MJ/kg | 12.2296 | 12.2299 | 12.2347 |  |  |

^1^ Each kilogram of the premix contains: 800–1.6 million IU vitamin A acetate, 3.00 mg cyanocobalamin, 650–125 million IU vitamin D3, 5.50 × 103 mg nicotinamide, 9 × 103 mg α-tocopheryl acetate, 4.00 × 103 mg D-calcium pantothenate, 350 mg menadione, 17.5 mg D-biotin, 1.50 × 103 mg riboflavin, 0.18 × 103–7.00 × 103 mg copper, 0.75 × 104–2.00 × 104 mg iron, 3.75 × 103–7.5 × 103 mg manganese, 1.00 × 104–4.00 × 104 mg zinc, 50–100 mg selenium, 15.0–30.0 mg iodine, etc. The rest is limestone powder. 1 ‘8:1, 5:1, 3:1’: different n-6/n-3 fatty acid ratios diets.

^2^ Results of nutritional levels were presented by mean ± standard deviation (SD) (n = 3, number of replicates).

**Table S2.** PUFA composition of diets.

| **Item^1^** | **Values of Each Group (%)^2^** | | |
| --- | --- | --- | --- |
|  | **8:1** | **5:1** | **3:1** |
| PUFA | 52.708 | 51.767 | 52.636 |
| n-3 PUFA | 6.152 | 9.117 | 28.408 |
| n-6 PUFA | 46.556 | 42.650 | 13.581 |
| n-6/n-3 PUFA | 8 | 5 | 3 |

^1^ PUFA, polyunsaturated fatty acid;

^2^ 8:1, 5:1, 3:1: different n-6/n-3 PUFA ratio diets.

**1.2 Supplementary Figures**


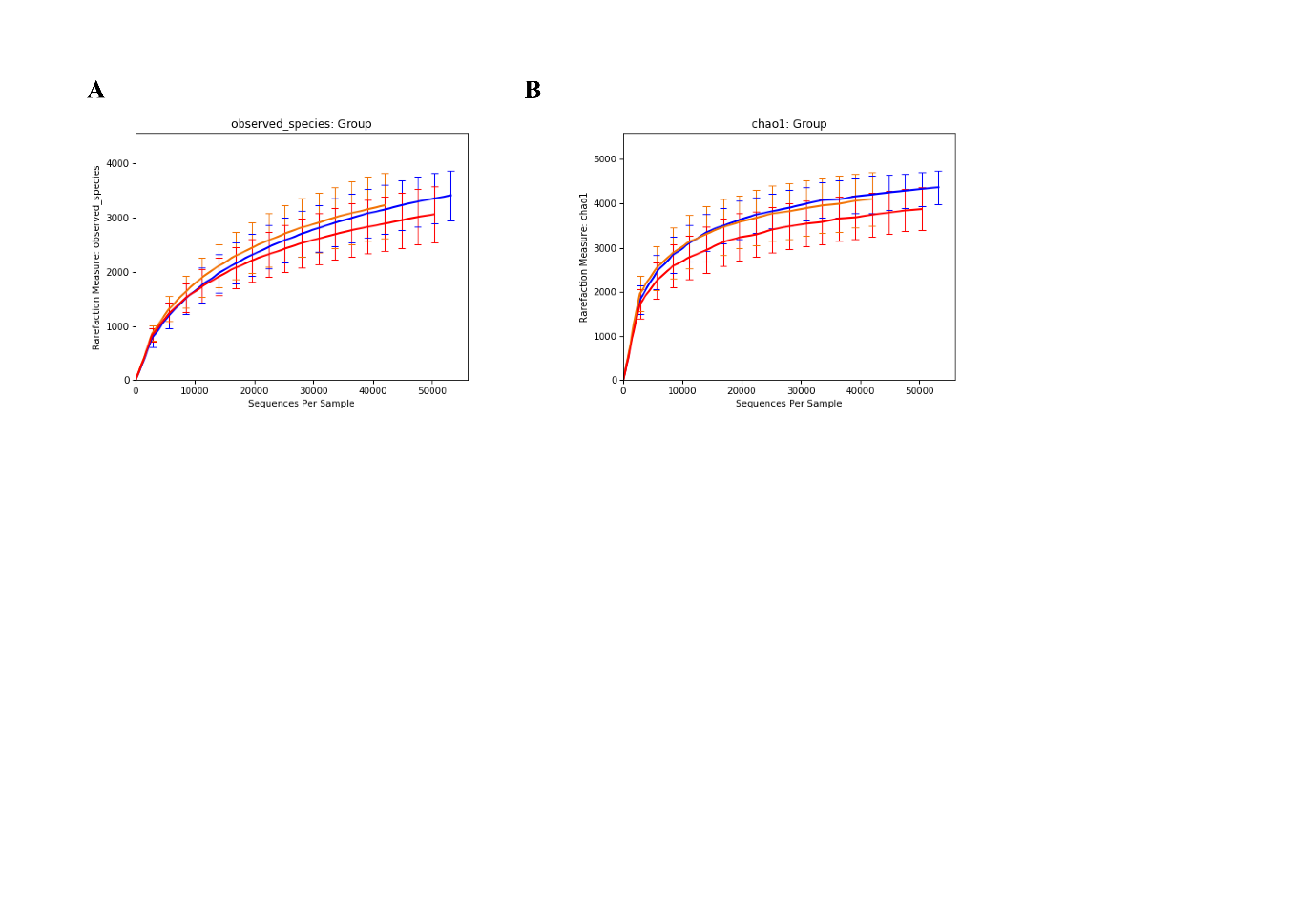


**Supplementary Figure 1.** Rarefaction curve comparing the number of OTUs found in the 16S rDNA gene libraries from microbiota in the colon.


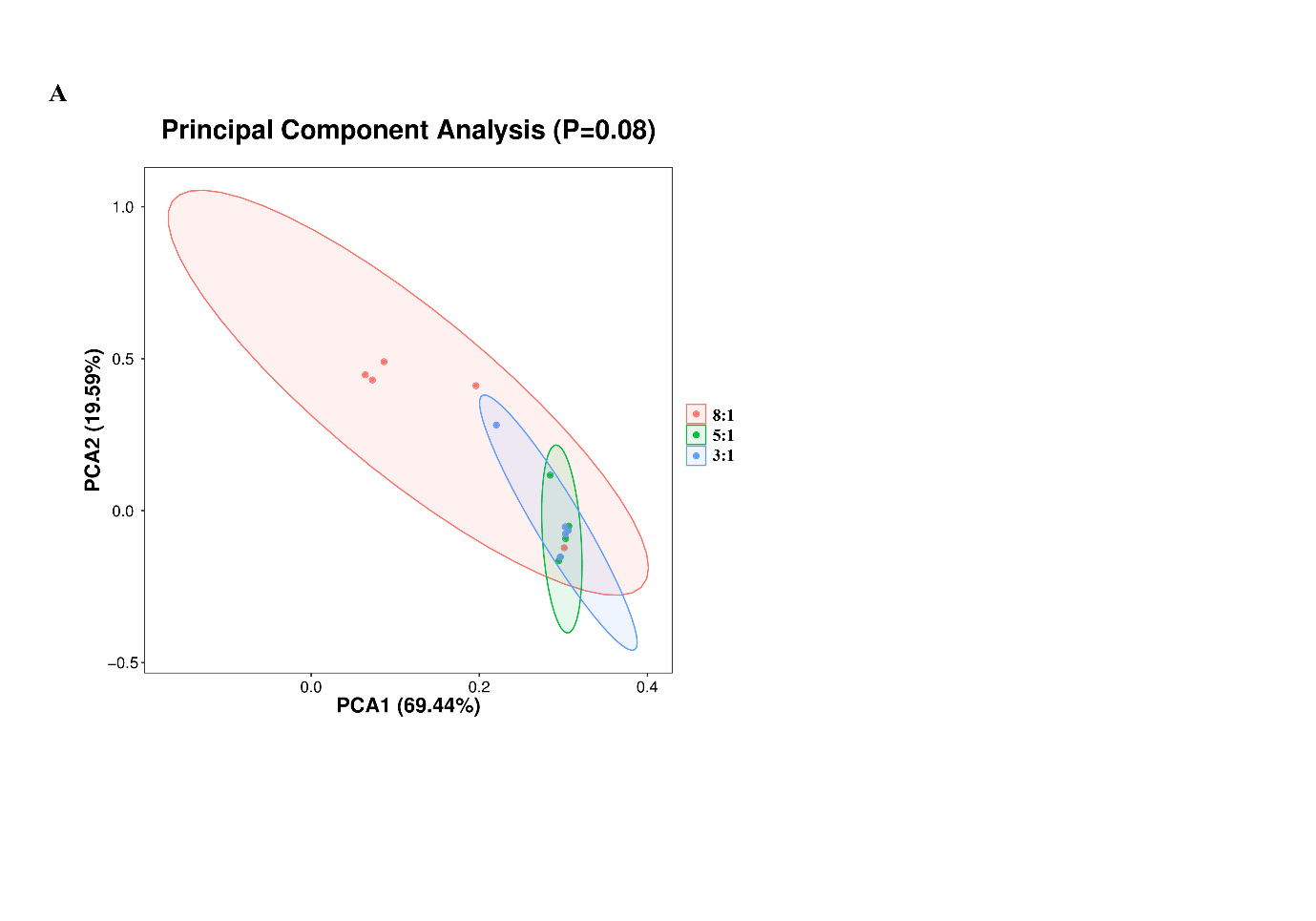


**Supplementary Figure 2.** Principal coordinates analysis (PCA) plot of differentially bacterial taxa in different group.


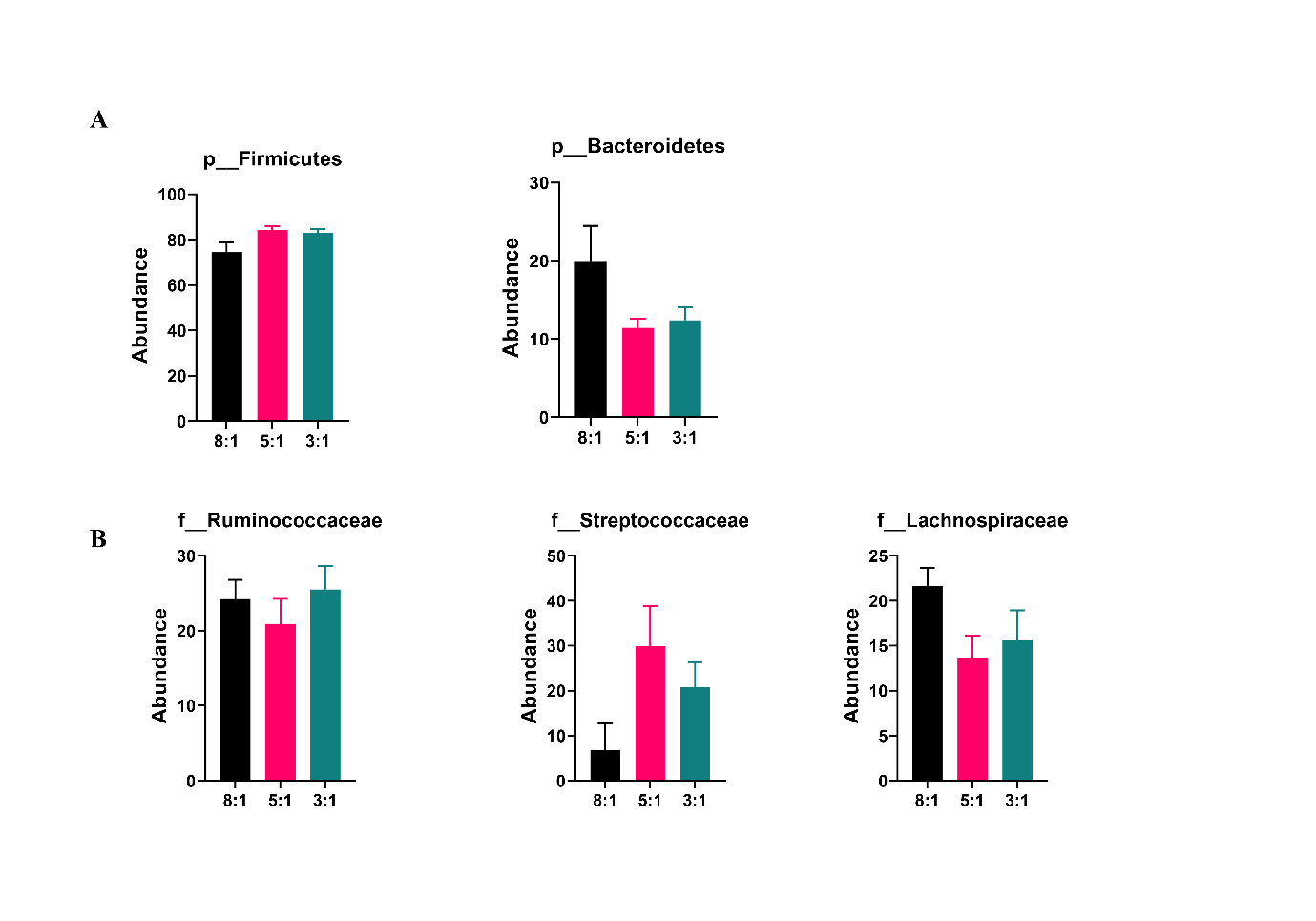


**Supplementary Figure 3.** The relative abundance of bacteria in colonic digesta of Heigai pigs at the phylum and family level. (**A**) Differential bacteria in colonic digesta at the phylum level. (**B**) Differential bacteria in colonic digesta at the family level.


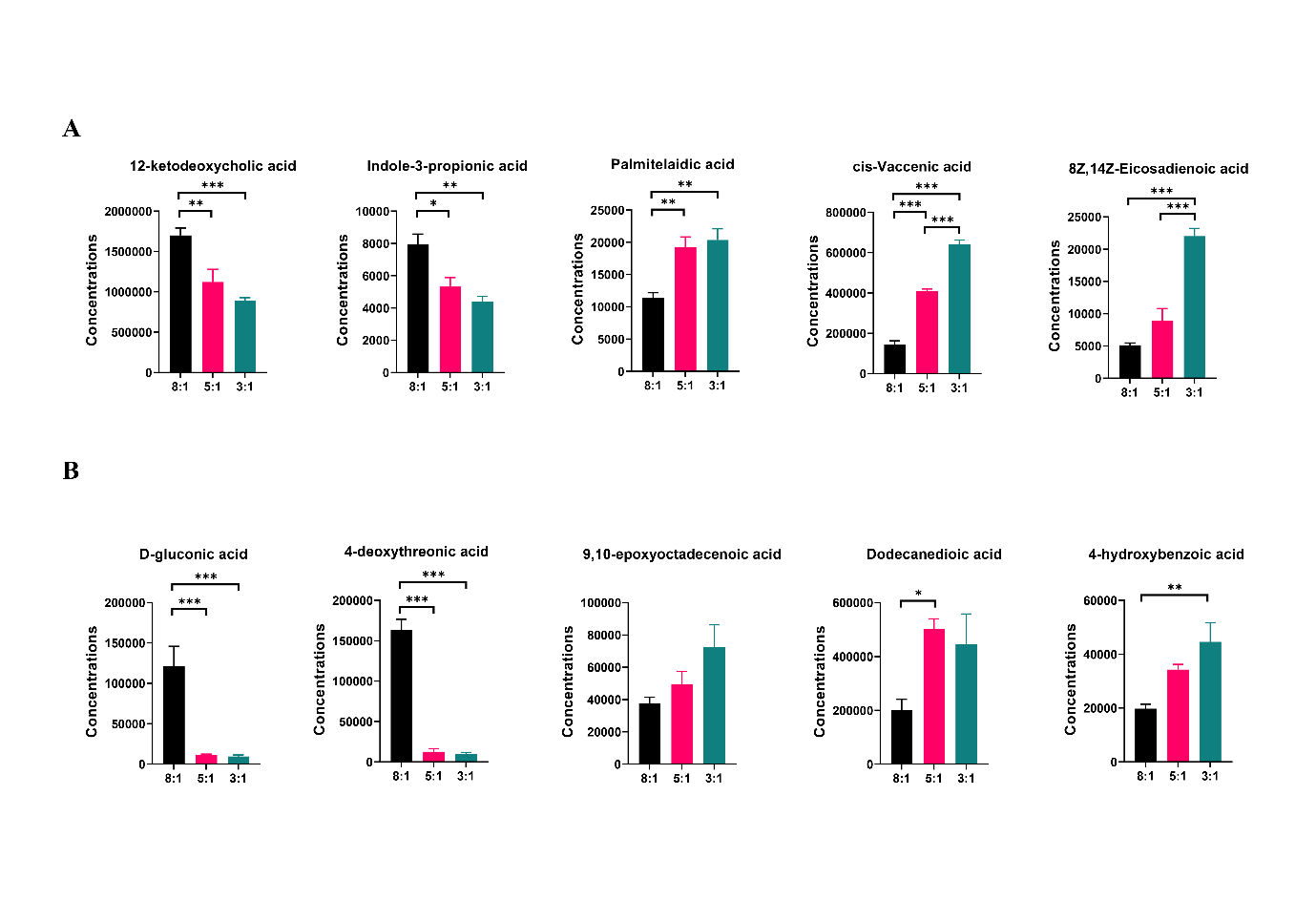


**Supplementary Figure 4.** Concentrations of differentially metabolites in different group. **(A)** ESI+ modes. **(B)** ESI- modes.
